# It takes a village: decreasing inappropriate antibiotic prescribing for upper respiratory tract infections

**DOI:** 10.1017/ash.2024.56

**Published:** 2024-04-29

**Authors:** Jamilah L. Shubeilat, Dan Ilges, Angie N. Ton, Maria Teresa A. Seville

**Affiliations:** 1 Division of Infectious Diseases, Mayo Clinic, Phoenix, Arizona, USA; 2 Department of Pharmacy Services, Mayo Clinic, Phoenix, Arizona, USA; 3 Department of Family Medicine, Mayo Clinic, Phoenix, Arizona, USA

## Abstract

**Objective:**

Prescribing of antibiotics for viral upper respiratory infections (URIs) remains a pressing public health problem. We sought to reduce inappropriate prescribing of antibiotics for URIs in Mayo Clinic Arizona.

**Design:**

Single-center, quasi-experimental, and retrospective cohort study

**Setting:**

Emergency medicine and all primary care departments

**Methods:**

The interventions included sharing baseline prescribing data, education, resources, and quarterly peer comparison reports. Encounters with diagnostic codes for respiratory infections commonly caused by viruses were categorized as Tier 3 (ie, never appropriate to prescribe antibiotics). Our goal was to reduce inappropriate prescribing for Tier 3 encounters by 22% in 2022.

**Results:**

Department education was completed by June 2022. The annual antibiotic prescribing rate for Tier 3 encounters was reduced by 29%, from a baseline rate of 23.6% in 2021 to 16.4% in 2022 (*P* < .001). The posteducation prescribing rate was 13.1%. Repeat respiratory-related healthcare contact within 14 days of Tier 3 encounters did not differ between patients prescribed and not prescribed an antibiotic in all of 2022 (4.7% antibiotic vs 4.2% no antibiotic, *P* = .595) or during the posteducation period (3.7% vs 4.6%, *P* = .604).

**Conclusion:**

A multi-faceted intervention, which included baseline education, syndrome-specific order panels, resources for symptomatic management, and peer comparison reports, resulted in significant reduction of inappropriate antibiotic prescribing for URIs.

## Introduction

The World Health Organization has identified antimicrobial resistance (AMR) as one of the greatest threats to global health.^1^ In 2016, there were an estimated 4.95 million deaths associated with AMR, about 1.3 million of which were directly attributable to resistant infections.^2^ This number is expected to reach around 10 million deaths per year worldwide by the year 2050.^2^ Antimicrobial stewardship efforts have historically focused on the inpatient setting. However, up to 85%–95% of all antibiotics are prescribed in outpatient settings.^3^ At least 30% of those prescriptions are considered inappropriate or unnecessary.^4^ In 2016, the Centers for Disease Control and Prevention published the Core Elements of Outpatient Antibiotic Stewardship, which encourages antimicrobial stewardship programs to identify one or more outpatient high-priority targets.^5^ The majority of outpatient antibiotics have been prescribed for upper respiratory tract infections.^4^ Therefore, we sought to reduce inappropriate prescribing of antibiotics for URIs at outpatient practices in Mayo Clinic Arizona (MCA).

## Methods

This single-center, quasi-experimental, retrospective cohort study was conducted at MCA in Phoenix, Arizona between 1 January 2021 and 31 December 2022. We divided respiratory ICD-10 codes into three tiers, including Tier 1 (“always prescribe antibiotics,” eg, pneumonia), Tier 2 (“sometimes prescribe,” eg, otitis media), and Tier 3 (“never prescribe,” eg, bronchitis), as previously reported (see supplemental material).^6^ The departments included were Family Medicine, Community Internal Medicine, Emergency Medicine, and Women’s Health Internal Medicine. We included all patient encounters with a Tier 3 primary diagnosis without secondary Tier 1 or 2 diagnoses. COVID-19-related diagnoses were excluded. We used an Epic dashboard (SlicerDicer) for data tracking.

**Figure 1.**
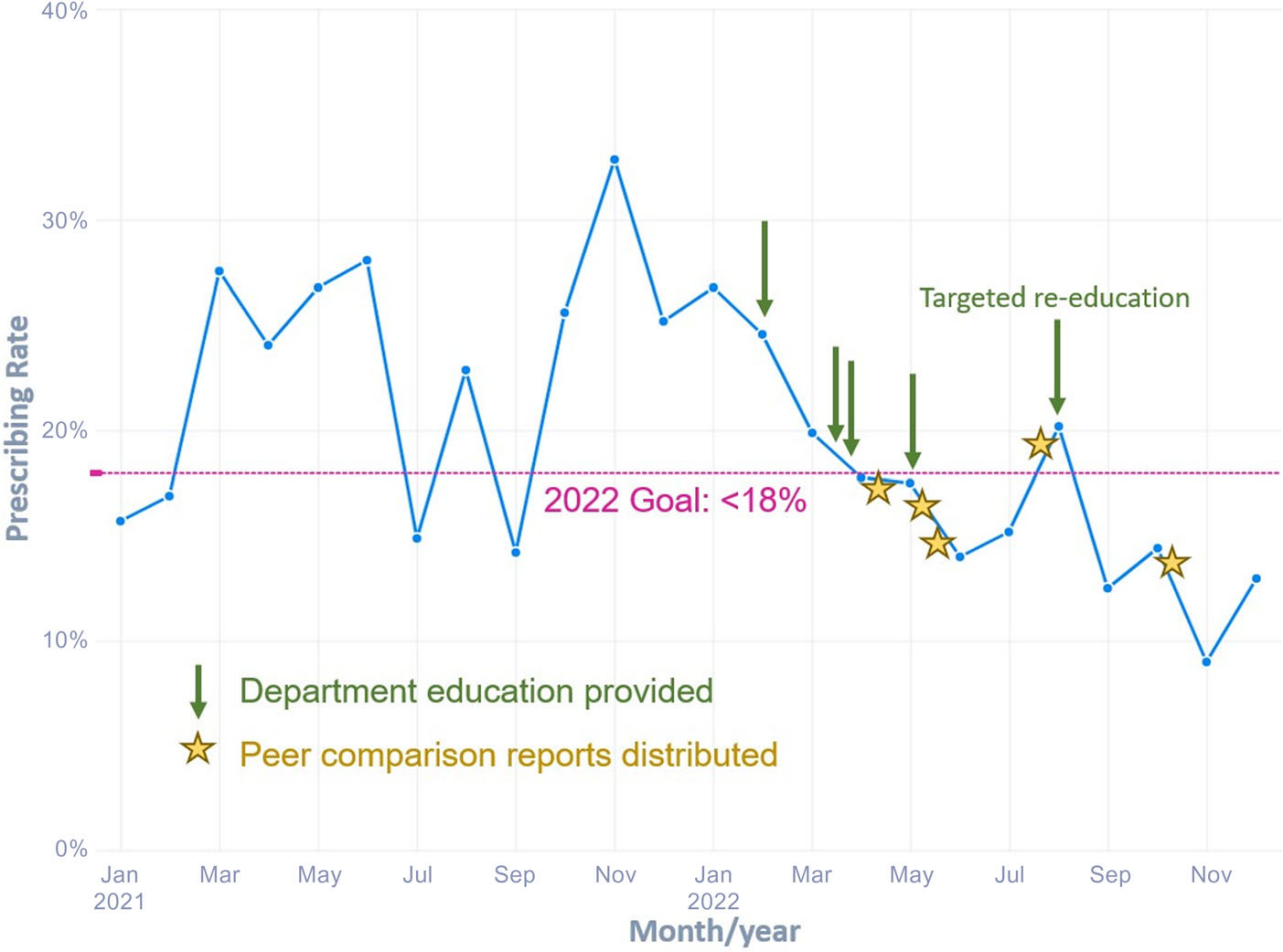
Prescribing rate for tier 3 encounters over time.

This project was conducted by a multidisciplinary antimicrobial stewardship team as part of routine quality improvement efforts. Our goal was to reduce inappropriate prescribing for Tier 3 respiratory encounters by 22% in 2022, from a baseline rate of 23.6% to a rate of 18.0%, which was the median rate for Mayo Clinic sites. Baseline data was collected from the preintervention period, spanning 1 January 2021 to 31 December 2021. Interventions began in 2022. We briefed each department on the project at their department meetings, including baseline departmental prescribing data, education on appropriate indications for antibiotics, patient-centered strategies for reducing antibiotic use, and a review of electronic resources developed specifically for the project. The resources included a syndromic ambulatory order panel (EZ ID Respiratory Order Panel) and a viral “prescription pad,” which contains simplified over-the-counter recommendations for symptomatic management of viral URIs, as well as patient education.^6,7^ Providers were also shown how to optimize Epic preferences to Tier 1 and 2 diagnoses to facilitate selection of the appropriate diagnoses. Additionally, we provided quarterly peer comparison reports (sample in supplemental material) to the department chairs and site leads (Figure 1). Focused re-education was provided based on provider prescribing performance. This study was deemed exempt from IRB approval.

Outcomes were compared between January to December 2021 (baseline) and January to December 2022 (intervention) periods. The Mayo Clinic Enterprise Antimicrobial Stewardship Program has annual data goals by calendar year hence January to December 2022 was included as the postintervention period; data from June to December 2022 after education was completed was analyzed separately to include the actual post-intervention period. The primary outcome was the rate of antibiotic prescribing for a Tier 3 diagnosis. The secondary outcome was the rate of 14-day repeat respiratory-related healthcare contact, defined as clinic, emergency, or hospital visit within 14 days for any respiratory diagnosis. We also evaluated the frequency of use of the EZ ID Respiratory Order Panel. Statistical analyses were performed using Chi-square or Fisher’s Exact tests for categorical variables and Student *t* test or Wilcoxon Rank Sum for continuous variables. A two-sided α level of 0.05 was used. All data was analyzed using IBM SPSS Statistics (Version 28.0.0.0).

## Results

**Figure 2.**
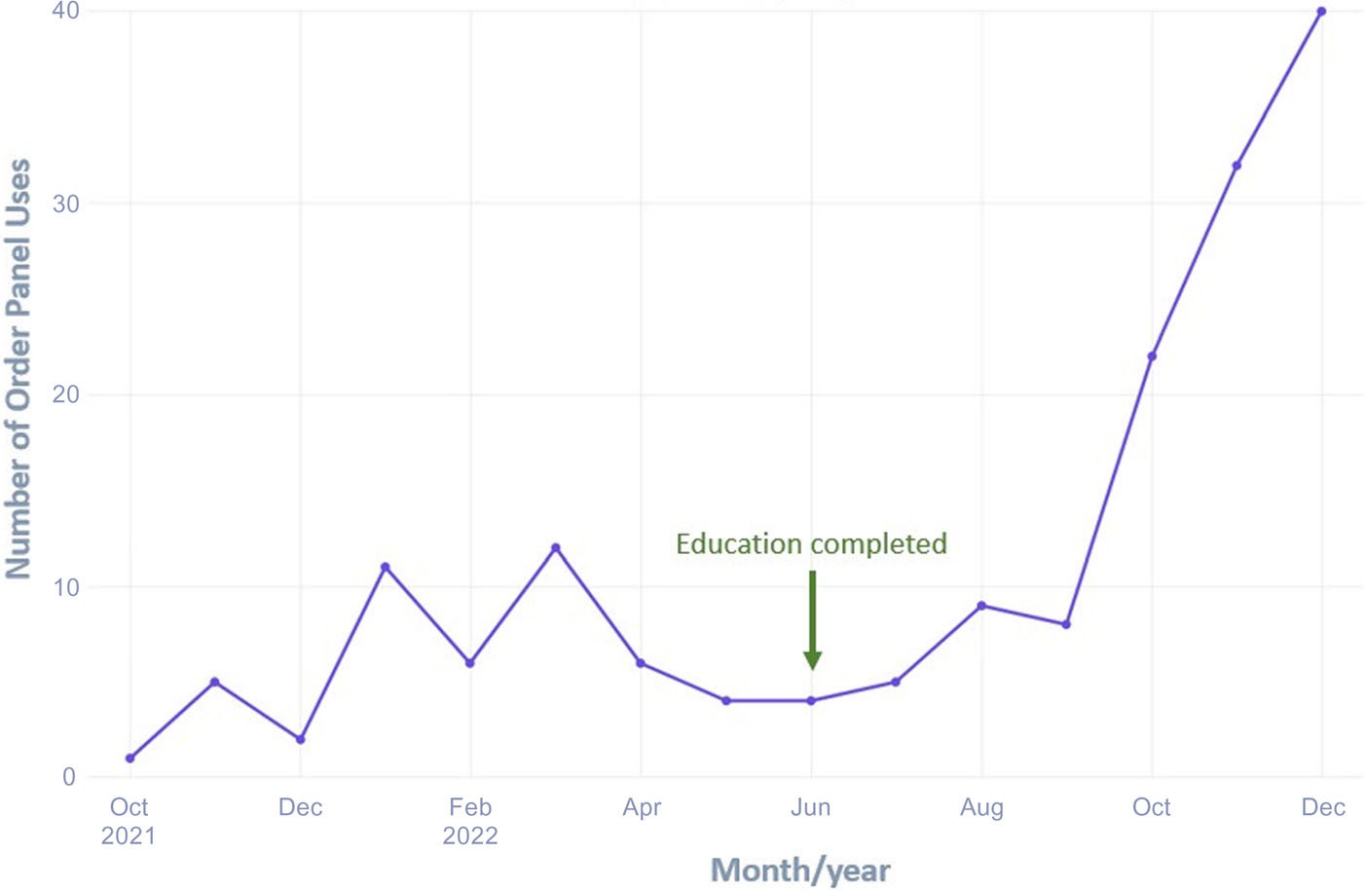
Number of Encounters using EZ ID respiratory order panel.

**Figure 3.**
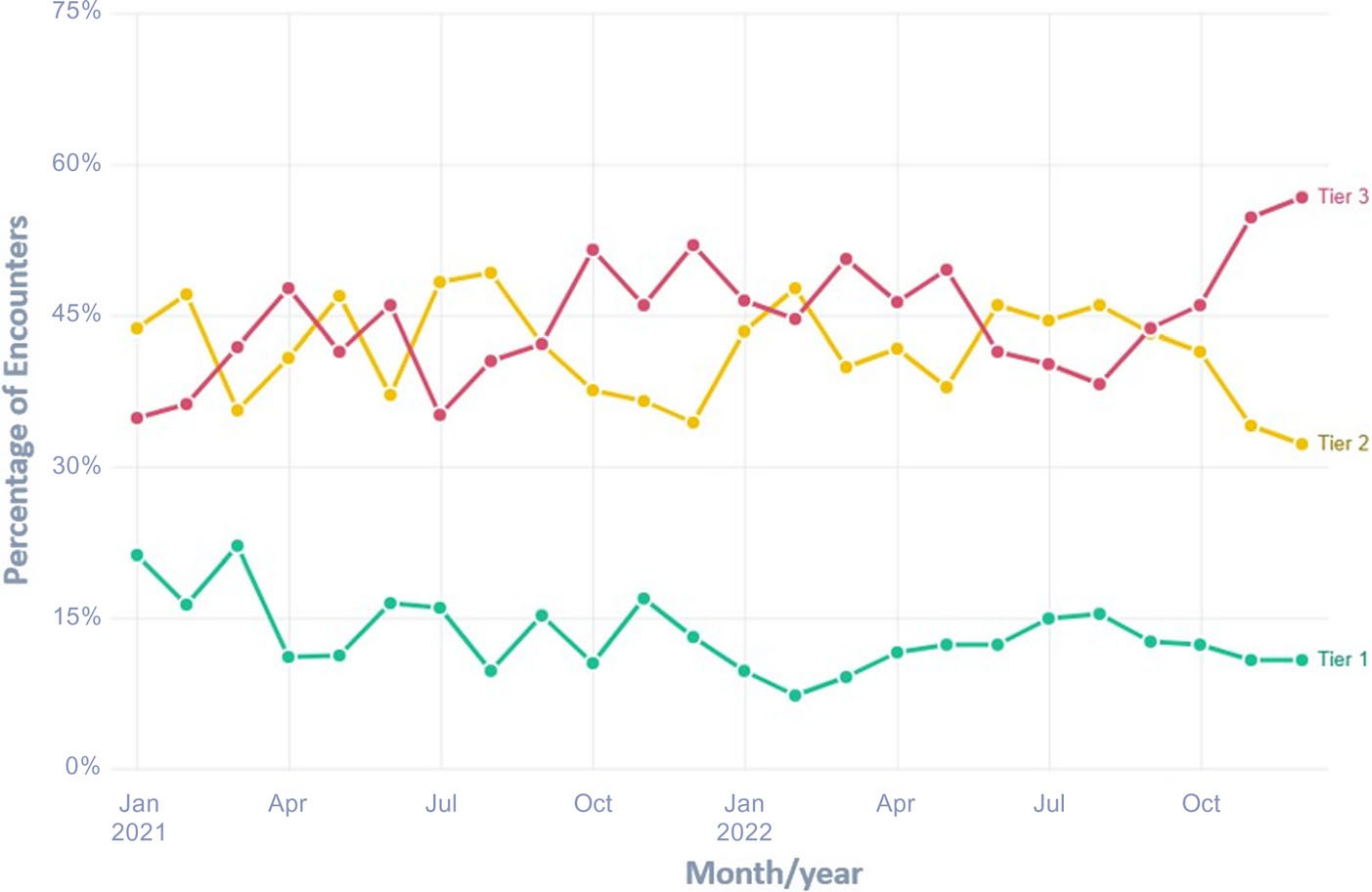
Percentage of encounters by respiratory diagnosis tiers over time. Figure 3. Legend: Figure shows the relative percentage of each respiratory infection diagnosis Tier over time, from January 2021 to December 2022. There was no increase in Tier 1 and Tier 2 diagnoses nor decrease in Tier 3 diagnoses over the course of 2022 compared with 2021.

We completed the intended department education by June of 2022 (Figure 1). A total of 3337 encounters met the inclusion criteria, including 1283 in 2021 and 2054 in 2022. Demographic data are summarized in Table 1. The annual antibiotic prescribing rate for Tier 3 encounters was reduced by 29%, from a baseline rate of 23.6% from January to December 2021 to 16.4% from January to December 2022 (*P* < .001) (Table 2). The posteducation prescribing rate from June 2022–December 2022 was 13.0%. Utilization of the EZ ID ambulatory order panel increased from an average of 1.5 uses per month in 2021 to 13.3 uses per month in 2022 (Figure 2). Repeat respiratory-related healthcare contact within 14 days of Tier 3 encounters did not differ significantly among patients who were prescribed antibiotics in comparison to those who were not in all of 2022 (4.7% antibiotic vs 4.2% no antibiotic, *P* = .595) or during the post-education period (3.7% vs 4.6%, *P* = .604) (Table 2). There was no appreciable diagnostic shift (eg, increase in Tier 1 and Tier 2 diagnoses with corresponding decrease in Tier 3 diagnoses) over the course of 2022 compared with 2021 (Figure 3).

**Table 1.** Baseline characteristics for tier 3 respiratory infection encounters

Characteristic	2021 (n=1283)	2022 (n=2054)
Age, mean (SD)	50.1 (21.0)	52.4 (21.4)
Age < 18 years	106 (8.3)	149 (7.3)
Female	815 (63.5)	1262 (61.4)
**Provider Type**		
Advanced Practice Provider	296 (23.1)	527 (25.7)
Fellow/resident	24 (1.9)	45 (2.2)
Physician	963 (75.1)	1482 (72.2)
**Department**		
Community Internal Medicine	281 (21.9)	412 (20.1)
Emergency Medicine	452 (35.2)	822 (40.0)
Family Medicine	487 (38.0)	740 (36.0)
Women’s Health	63 (4.9)	80 (3.9)
**Encounter Type**		
Inperson	940 (73.3)	1606 (78.2)
Virtual	343 (26.7)	448 (21.8)
**Diagnosis**		
Bronchitis/bronchiolitis	221 (17.2)	271 (13.2)
Influenza	36 (2.8)	325 (15.8)
Laryngitis/pharyngitis	46 (3.6)	55 (2.7)
Rhinitis	1 (0.1)	3 (0.1)
Serous otitis media/ear disorders	328 (25.6)	346 (16.8)
URI, unspecified	601 (46.8)	79 (3.8)
Specific antibiotics prescribed*		
Amoxicillin	17 (5.6)	24 (7.1)
Amoxicillin/clavulanate	39 (12.9)	60 (17.8)
Azithromycin	176 (58.1)	155 (46.0)
Cefdinir	10 (3.3)	10 (3.0)
Cefuroxime	1 (0.3)	4 (1.2)
Clarithromycin	1 (0.3)	0
Doxycycline	63 (20.8)	83 (24.6)
**Levofloxacin**	4 (1.3)	4 (1.2)
**Penicillin VK**	0	88 (4.3)

Data is shown as number (%) unless specified.

* Some encounters generated more than one prescription, n=311 in 2021, n=340 in 2022.

**Table 2.** Antibiotic prescribing rate and repeat respiratory-related healthcare contact by year and antibiotic prescription

Outcomes	2021	2022	***P*** **-value**
**Tier 3 Prescribing Rate**			
January-December 2022	303/1283 (23.6)	337/2054 (16.4)	<0.001
June-December 2022 (posteducation)	303/1283 (23.6)	161/1239 (13.0)	<0.001
**Repeat Respiratory-Related Healthcare Contact**	**Antibiotic**	**No Antibiotic**	
January-December 2022	16/337 (4.7)	72/1717 (4.2)	0.595
June-December 2022 (posteducation)	6/161 (3.7)	50/1078 (4.6)	0.604

Data is shown as number (%).

## Discussion

Prior studies have identified high rates of antibiotic prescribing for nonbacterial upper respiratory tract infections with rates reaching up to 50% in the general population and up to around 70% among US veterans.^8^ Therefore, we recognized antibiotic prescribing for URIs as a crucial outpatient stewardship target for our center.

Our active intervention was multi-faceted and targeted multiple key elements that we identified as essential to decreasing prescribing rates. We provided baseline and distributed peer comparison reports to department leaders that helped each department identify prescribing patterns that could be improved, for which as-needed education was provided. Utilization of the EZ ID ambulatory order panel increased from an average of 1.5 uses per month in 2021 to 13.3 uses per month in 2022. These syndrome-specific order panels in the electronic medical record list appropriate non-antibiotic interventions for different respiratory diagnoses which may have helped reduce prescribing rates. Similarly, the viral prescription pad, which contains appropriate non-antibacterial therapies that patients can use for symptomatic relief may have helped reduce patient demand for antibiotics.^9^ The individual contribution of each of the measures cannot be determined, but when used in combination, these interventions significantly reduced the inappropriate prescribing of antibiotics for viral upper respiratory tract infections. Similar findings were observed at the Mayo Clinic Enterprise level.^6^

Our study had limitations. First, we were unable to account for delayed (ie, watch and wait) prescribing or prescribing outside of a provider visit. Second, antibiotics prescribed in the outpatient setting within our system did not require an associated diagnosis, which limited the information we had about such prescriptions. Third, our Tier 3 diagnoses included a diagnosis of URI not otherwise specified; it is possible that antibiotics may have been appropriate for some of these encounters. Fourth, we are unable to completely account for the possibility of providers avoiding Tier 3 diagnoses to avoid detection, although there did not appear to be appreciable shift in diagnosis tiers (figure 3). Fifth, we excluded all COVID-19-related diagnoses as antimicrobial prescribing rate for ambulatory COVID-19 patients can be as low as 3%, and inclusion of these encounters could artificially reduce the URI prescribing rate.^10^ Finally, the SlicerDicer model included only respiratory infection codes and antibiotics typically prescribed for respiratory infections, but other indications for antibiotics such as a urinary tract infection were not excluded. It is thus possible that some patients with a Tier 3 primary diagnosis who received an antibiotic prescription for a secondary diagnosis of another bacterial infection (eg, cellulitis, urinary tract infection) were incorrectly identified as inappropriate; however, a prior validation of the model demonstrated that this phenomenon occurred rarely. Moreover, the exclusion of other indications for antibiotics would have decreased the inappropriate antibiotic prescribing rate for Tier 3 respiratory infections further.

In conclusion, a multi-faceted intervention, which included baseline education, promotion of syndrome-specific order panels, dissemination of resources for symptomatic management, and distribution of quarterly peer comparison reports, resulted in significant reduction of inappropriate antibiotic prescribing for URIs.

## Supporting information

Shubeilat et al. supplementary material Shubeilat et al. supplementary material

## Supplementary material

For supplementary material accompanying this paper visit http://doi.org/10.1017/ash.2024.56. click here to view supplementary material
