# Supplementary material for: It takes a village: decreasing inappropriate antibiotic prescribing for upper respiratory tract infections
Source: Antimicrob Steward Healthc Epidemiol. 2024 Apr 29;4(1):e63. doi: 10.1017/ash.2024.56 (PMC11062784; doi:10.1017/ash.2024.56)

**It Takes a Village: Decreasing Inappropriate Antibiotic Prescribing for Upper Respiratory Tract Infections**

**Ref: MS no. ASHE-D-23-00277**

**Supplemental material**

**Diagnostic Tiers with Example Diagnoses**

| Tier 1 (Always prescribe antibiotics) | Tier 2 (Sometimes prescribe) | Tier 3 (Never prescribe) |
| --- | --- | --- |
| Bacterial Pneumonia  Pertussis  Streptococcal Pharyngitis | Acute Pharyngitis  Acute Laryngopharyngitis  Suppurative Otitis Media  Acute Sinusitis  COPD Exacerbations | Bronchitis  Croup  Influenza  Allergic Rhinitis  Serous Otitis Media |

**Example Peer Comparison Report**


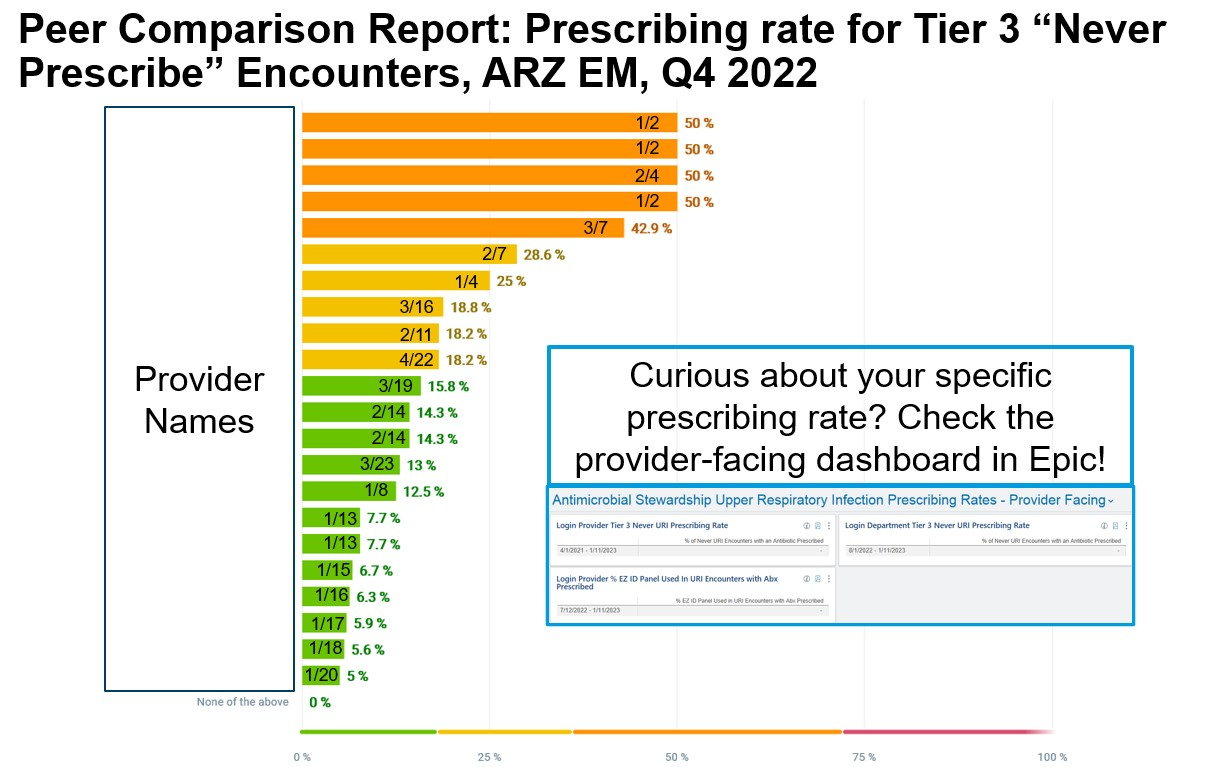

Supplement: Shubeilat et al. supplementary material [file S2732494X24000561sup001.docx]
